# Supplementary material for: PRAME expression in melanoma is negatively regulated by TET2-mediated DNA hydroxymethylation
Source: bioRxiv. 2024 Jul 26:2024.07.26.605293. Preprint. [Version 1] doi: 10.1101/2024.07.26.605293 (PMC11291125; doi:10.1101/2024.07.26.605293)

bioRxiv preprint doi: https://doi.org/10.1101/2024.07.26.605293; this version posted July 26, 2024. The copyright holder for this preprint (which was not certified by peer review) is the author/funder, who has granted bioRxiv a license to display the preprint in perpetuity. It is made available under aCC-BY 4.0 International license.

SUPPLEMENTARY MATERIALS

Supplementary Table 1. Summary of IF and IHC staining of nevus and melanoma patient samples

Staining	Diagnosis	PRAME%	5hmC_score	differential PRAME alone (a,c)	differential dual biomarkers (a,b,c)
	**27 nevus**	**0**	**12**	**PRAME-, benign**	**PRAME- AND 5hmC+, benign**
IHC	nevus	50	4	PRAME-, benign	PRAME-/5hmC- , melanoma
IHC	nevus	30	12	PRAME-, benign	PRAME- AND 5hmC+, benign
IHC	nevus	20	9	PRAME-, benign	PRAME- AND 5hmC+, benign
	**12 MIS**	**>= 90%**	**0**	**PRAME+, melanoma**	**PRAME+ OR 5hmC-, meanoma**
IF	MIS	95	6	PRAME+, melanoma	PRAME+ OR 5hmC-, meanoma
IHC	MIS	80	4	PRAME+, melanoma	PRAME+ OR 5hmC-, meanoma
IHC	MIS	80	3	PRAME+, melanoma	PRAME+ OR 5hmC-, meanoma
IF	MIS	50	10	PRAME-, benign	PRAME-/5hmC+ benign
IHC	MIS	30	2	PRAME-, benign	PRAME+ OR 5hmC-, meanoma
IHC	MIS	0	9	PRAME-, benign	PRAME-/5hmC+ benign
IHC	MIS	0	9	PRAME-, benign	PRAME-/5hmC+ benign
	**33 primary MM**	**>= 90%**	**0**	**PRAME+, melanoma**	**PRAME+ OR 5hmC-, meanoma**
IF	primary MM	80	4	PRAME+, melanoma	PRAME+ OR 5hmC-, meanoma
IHC	primary MM	50	1	PRAME-, benign	PRAME+ OR 5hmC-, meanoma
IHC	primary MM	30	3	PRAME-, benign	PRAME+ OR 5hmC-, meanoma
IHC	primary MM	1	9	PRAME-, benign	PRAME-/5hmC+ benign
IHC	primary MM	0	9	PRAME-, benign	PRAME-/5hmC+ benign
	**23 Met**	**>= 90%**	**0**	**PRAME+, melanoma**	**PRAME+ OR 5hmC-, meanoma**
IF	MET	95	4	PRAME+, melanoma	PRAME+ OR 5hmC-, meanoma

(a) $PRAME^+$, >=75% PRAME positive cells

(b) $5hmC^+$, score >=4

(c) highlight, staining differential inconsistent with diagnosis.

bioRxiv preprint doi: https://doi.org/10.1101/2024.07.26.605293; this version posted July 26, 2024. The copyright holder for this preprint (which was not certified by peer review) is the author/funder, who has granted bioRxiv a license to display the preprint in perpetuity. It is made available under aCC-BY 4.0 International license.

Supplementary Table 2. Gene-specific RT-qPCR primers

Gene name	Sequence	
PRAME	forward	5'-GGAGTGCTGATGAAGGGACAAC-3'
	reverse	5'-CAGTCCAGAAGTCCTGATGAGAG-3'
TET2	forward	5'-GCTTACCGAGACGCTGAGGAAA-3'
	reverse	5'-AGAGAAGGAGGCACCACAGGTT-3'
β-Actin	forward	5'-CACCATTGGCAATGAGCGGTTC-3'
	reverse	5'-AGGTCTTTGCGGATGTCCACGT-3'

bioRxiv preprint doi: https://doi.org/10.1101/2024.07.26.605293; this version posted July 26, 2024. The copyright holder for this preprint (which was not certified by peer review) is the author/funder, who has granted bioRxiv a license to display the preprint in perpetuity. It is made available under aCC-BY 4.0 International license.

Supplementary Table 3. Antibody list of CyCIF, IF and IHC

Antibody	Fluorophore	Clone	Vendor	Catalogue #	Staining
5hmC	CY3(ab6939)		Active Motif	39769	IF, IHC
PRAME	MouseIgG2a AF647(A21241)	CL5146	Invitrogen	MA5-31408	IF, IHC
MART-1	MouseIgG2b AF488(A21141)	M2-7C10	Biolegend	917902	IF
goat anti-rabbit IgG	Alexa Fluor 488-green		Invitrogen	A11008	IF
goat anti-rabbit IgG	Alexa Fluor 647		Invitrogen	A21245	IF
goat anti-mouse IgG2b	Alexa Fluor 488-green		Invitrogen	A21141	IF
TET2	Alexa Fluor 488-green		Proteintech	21207-AP	CyCIF,IF, IHC, WB
SOX10		SOX10/1074	Abcam	ab216020	CyCIF
Goat Anti-Mouse IgG1-AF647	Alexa Fluor 647		Jackson ImmunoResearch, Inc.	115-605-205	CyCIF
MART-1-AF647	Alexa Fluor 647	EPR20380	Abcam	ab225500	CyCIF

bioRxiv preprint doi: https://doi.org/10.1101/2024.07.26.605293; this version posted July 26, 2024. The copyright holder for this preprint (which was not certified by peer review) is the author/funder, who has granted bioRxiv a license to display the preprint in perpetuity. It is made available under aCC-BY 4.0 International license.

Supplementary Figures

SF1. Multiplex immunofluorescence staining of nevus/melanoma tissue microarray.

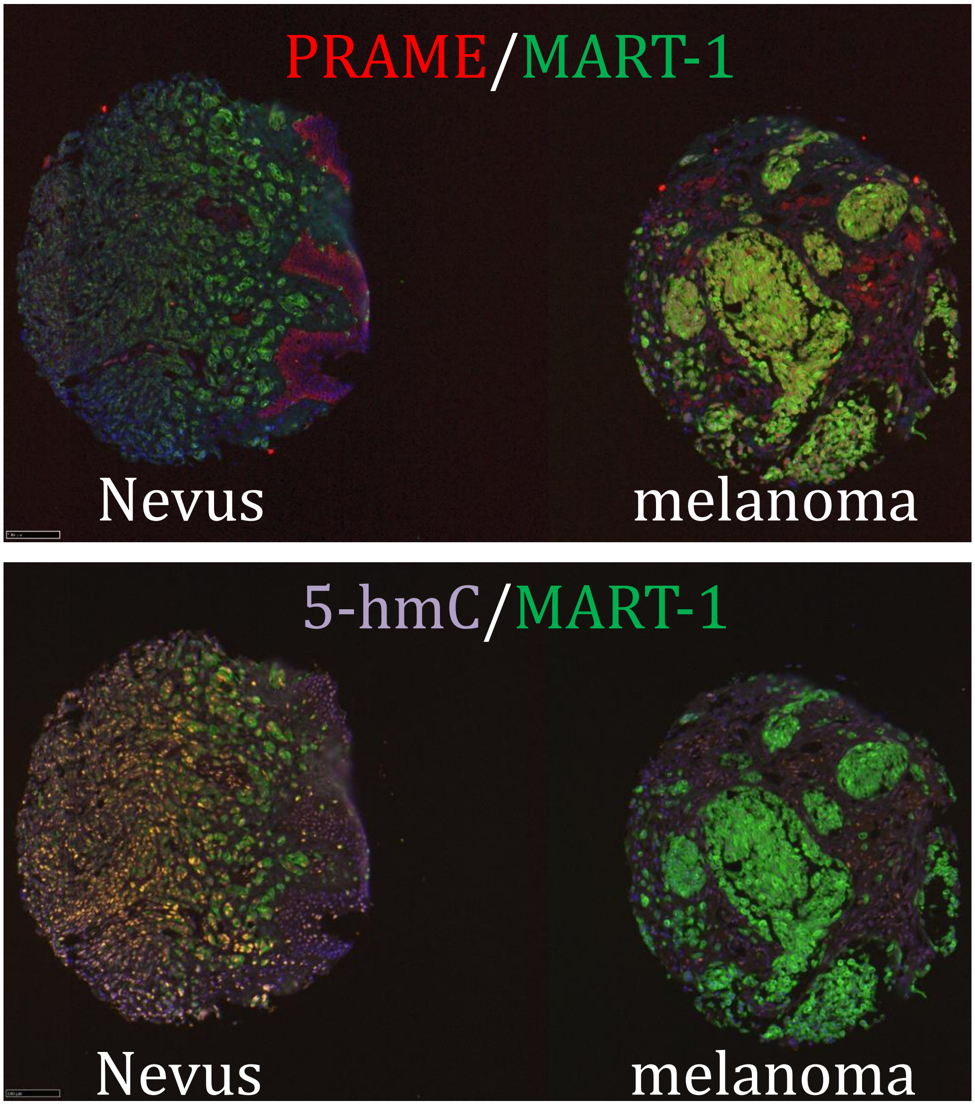

bioRxiv preprint doi: https://doi.org/10.1101/2024.07.26.605293; this version posted July 26, 2024. The copyright holder for this preprint (which was not certified by peer review) is the author/funder, who has granted bioRxiv a license to display the preprint in perpetuity. It is made available under aCC-BY 4.0 International license.

SF2. Multiplex immunofluorescence staining of nevus/melanoma tissue specimens.

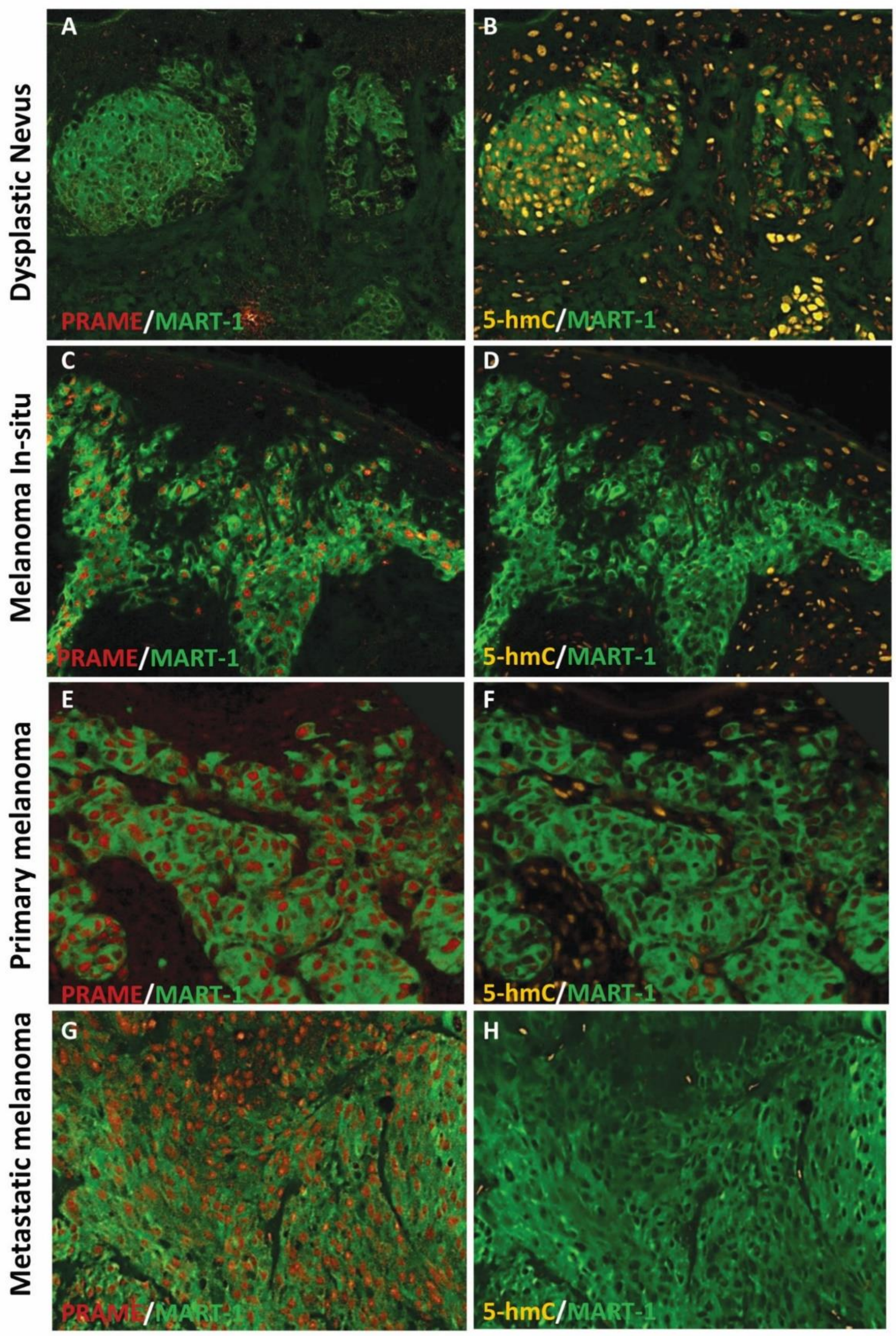

Supplement: Supplement 1 [file NIHPP2024.07.26.605293v1-supplement-1.pdf]
